# A Comparison of Molecular Techniques for Improving the Methodology in the Laboratory of Pharmacogenetics

**DOI:** 10.3390/ijms252111505

**Published:** 2024-10-26

**Authors:** María Celsa Peña-Martín, Elena Marcos-Vadillo, Belén García-Berrocal, David Hansoe Heredero-Jung, María Jesús García-Salgado, Sandra Milagros Lorenzo-Hernández, Romain Larrue, Marie Lenski, Guillaume Drevin, Catalina Sanz, María Isidoro-García

**Affiliations:** 1 Department of Clinical Biochemistry, University Hospital of Salamanca, 37007 Salamanca, Spain; mariacelsa@usal.es (M.C.P.-M.); emarcosv@saludcastillayleon.es (E.M.-V.); mbgarcia@saludcastillayleon.es (B.G.-B.); dhheredero.ibsal@saludcastillayleon.es (D.H.H.-J.); mjgarciasal@saludcastillayleon.es (M.J.G.-S.); smlorenzo@saludcastillayleon.es (S.M.L.-H.); misidoro@saludcastillayleon.es (M.I.-G.); 2 Pharmacology-Toxicology and Pharmacovigilance Department, Angers University Hospital, F-49100 Angers, France; guillaume.drevin@chu-angers.fr; 3 Institute for Biomedical Research of Salamanca, 37007 Salamanca, Spain; 4 CNRS, Inserm, CHU Lille, UMR9020-U1277—CANTHER—Cancer Heterogeneity Plasticity and Resistance to Therapies, University of Lille, F-59000 Lille, France; romain.larrue@chu-lille.fr; 5 CHU Lille, Institut Pasteur de Lille, ULR 4483, IMPECS-IMPact of the Chemical Environment on Health, University of Lille, F-59000 Lille, France; marie.lenski@univ-lille.fr; 6 Department of Microbiology and Genetics, University of Salamanca, 37007 Salamanca, Spain; 7 Department of Medicine, University of Salamanca, 37007 Salamanca, Spain

**Keywords:** pharmacogenetics, polypharmacy, precision medicine, microarray, mass spectrometry, real-time PCR

## Abstract

One of the most critical goals in healthcare is safe and effective drug therapy, which is directly related to an individual’s response to treatment. Precision medicine can improve drug safety in many scenarios, including polypharmacy, and it requires the development of new genetic characterization methods. In this report, we use real-time PCR, microarray techniques, and mass spectrometry (MALDI-TOF), which allows us to compare them and identify the potential benefits of technological improvements, leading to better quality medical care. These comparative studies, as part of our pharmacogenetic Five-Step Precision Medicine (5SPM) approach, reveal the superiority of mass spectrometry over the other methods analyzed and highlight the importance of updating the laboratory’s pharmacogenetic methodology to identify new variants with clinical impact.

## 1. Introduction

The primary goal of patient care is to improve quality of life through safe and effective pharmacological intervention. Therefore, the application of genetics in the clinical care process is not limited to the diagnostic field but also affects the therapeutic aspects of care through pharmacogenetics.

Pharmacogenetics has been a growing area of interest since its inception [1], as it studies the impact of genes and genetic variation on drug ADME (absorption, distribution, metabolism, and excretion) and an individual’s response to a drug or drug combination. This individual drug response is based on the pharmacogenetic (PK) and pharmacodynamic (PD) properties of medication and the complex interactions caused by the combination of drug–drug interactions (DDIs) and drug–gene interactions (DGIs) [2], both increased by polypharmacy, also taking into account the possibility of multiple ligands [3,4]. In addition, other situations may be influencing an individual’s metabolizing capacity. In this regard, the existence of alternative metabolizing pathways, the possibility that a drug may act as a substrate, inhibitor, or inducer of a specific pathway, or the alteration of the actual ability to metabolize drugs due to non-genetic factors (inflammation, pregnancy, smoking, gender), leading to a phenoconversion phenomenon, are all important [5]. This situation also increases the occurrence of adverse events, as polypharmacy is becoming the rule rather than the exception as the population ages worldwide [6,7]. Therefore, pharmacogenetic studies can improve patient care and treatment outcomes, mainly when conducted before treatment initiation [8,9].

Drugs metabolized by isoenzymes from the cytochrome P450 (CYP450) are the most prevalent in clinical practice; so, genetic variations in these enzymes may affect the response to medication [10,11,12]. Genetic polymorphisms in enzymes encoded by the CYP1, CYP2, CYP3, and CYP4 families are commonly studied in clinical practice, as they contribute significantly to Phase I drugs and xenobiotics biotransformation [13]. Phase II enzymes and drug transporters such as ABCB1 can also contribute to treatment response [14].

Among the significant CYP isoforms responsible for the metabolism of most drugs, genetic variation and its contribution to enzyme activity are variable. In particular, *CYP2D6* is highly polymorphic, and its variants can cause a wide range of enzyme activity. The translation of genotype to phenotype is not standardized across clinical laboratories despite the published recommendations of the Clinical Pharmacogenetic Consortium (CPIC) [15,16]. It must be considered that the frequencies of those variants can be different depending on the various populations worldwide [17,18], although it should be noted that only a small sample of the world’s population has been studied [19]. In the case of *CYP2D6*, there are two pseudogenes (*CYP2D7*, *CYP2D8*) that pose a challenge in the process of determining its genotype [20,21], so it is also necessary to highlight the need for molecular technique improvement.

*CYP1A2* genotyping becomes especially relevant since it is a highly inducible gene, and tobacco is a potent inducer. Therefore, in smoking patients, it is essential to know the existing variants since tobacco interactions are widely underestimated concerning drug–drug interactions, and sudden cessation of consumption, as in the case of hospitalization, can significantly affect the variation in a patient’s response to treatment [22]. Likewise, caffeine intake can also interfere with metabolization by this enzyme, as can the pathological conditions of the patient, such as renal dysfunction [23,24,25].

Genotyping technologies have improved significantly in the last few decades. They can be used for various applications, including improving clinical care, as they can help us understand some diseases and patient response to different treatments [26].

Historically, a genotype was inferred from its expressed phenotype [27,28]. Subsequently, thanks to the discovery of the double-helix structure of DNA in 1953 [29] and its study, initially by techniques such as Southern blotting [30] or the Sanger sequencing method [31], phenotypes are expressed as a function of their genotype. This research led to the discovery of the Polymerase Chain Reaction (PCR), which allowed for the amplification of DNA fragments [32], enabling sequencing by laser-induced fluorescence measurement and capillary gel electrophoresis [33,34] and evolving into the development of DNA microarray technology [35]. This also decreased the time required for genotyping as it permits us to obtain a large number of variants simultaneously. The early 21st century was dedicated to developing Next-Generation Sequencing (second generation) methods, increasing sequencing data, and decreasing analysis costs [36]. Nowadays, high-throughput sequencing and short fragments are currently being replaced by long-read fragment sequencing (third generation sequencing), as it can sequence long strands of genetic material (DNA or RNA) in one go without breaking it up into smaller fragments [37]. Other techniques, such as mass spectrometry with end-point PCR, enable highly multiplexed reactions, providing a genetic analysis [38].

Considering the quick and extensive development of these new techniques, it is also necessary to consider updating the results given the new gene variants characterized that may interfere with the diagnosis and treatment of patients.

Therefore, the main objective of our work is to study various methods of pharmacogenetic analysis used in our Reference Unit of Pharmacogenomics and Precision Medicine to identify potential advantages of technological improvements that result in better quality medical care.

## 2. Results

More than 4000 pharmacogenetic determinations were performed in 596 patients in the Pharmacogenetics and Precision Medicine Reference Unit of the University Hospital of Salamanca.

### 2.1. Clinical Data Collection

**Figure 1:**
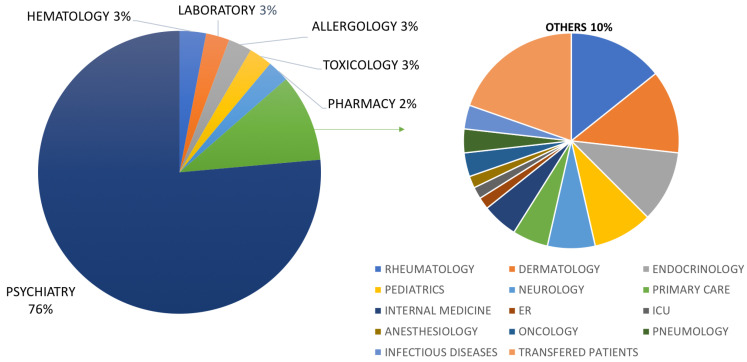
The distribution of patients according to their clinical service of origin.

The vast majority of the studied patients came from Psychiatry (428 patients), as we can observe in Figure 1, including subspecialties such as Child Psychiatry (12 patients) and the Eating Disorders Unit (24 patients). Other clinical services that have referred patients to the unit include Hematology, Toxicology, Allergy, Rheumatology, Dermatology, Endocrinology, Pediatrics, Neurology, Primary Care, Internal Medicine, Oncology, Pulmonology, Infectious Diseases, Emergency Medicine, the Intensive Care Unit (ICU), and Anesthesiology.

### 2.2. Pharmacogenetic Analysis

Regarding the patients analyzed, in the case of the first methodological approach (real-time PCR and microarray technique), 48.18% were women and 51.82% were men, with an average age of 42.87 ± 19.4 years, while in the case of the patients analyzed by MALDI-TOF, 46.57% were women and 53.43% were men, with an average age of 43.98 ± 18.6 years. There were no differences according to the age or sex of the patients studied.

Of the 4000 pharmacogenetic determinations performed, more than 1600 were through mass spectrometry MALDI-TOF, and the rest by using high-resolution melting and microarray technology.

The results were based on the metabolizing effect of the alleles and genotypes analyzed to evaluate the potential clinical impact of the patients under study. Table 1 shows the allelic frequencies in the population studied, while Table 2 shows the distribution of genotype frequencies in our population.

There are no significant discrepancies (*p* < 0.05) between the results of the two techniques concerning the decreased or increased inducible enzyme activity.

### 2.3. Comparison of Genetic Characterization Methods

A comparison of the results obtained from the genotyping of several genes in the same patient using different techniques was carried out on 657 occasions. Of these comparisons, 95.7% were concordant, compared to 4.3% of mismatches, most caused by improved genotyping technology. Those mismatches are distributed as shown in Table 3, where we can also observe whether the discordances produced a change in the phenotype of the patients studied.

Most mismatched cases that produce a change in phenotype were due to a more exhaustive study and using a greater variety of variants of the new methodology.

The two discordant cases were initially reported for *CYP1A2* as highly inducible metabolizers and showed differences in enzyme activity with the second analysis method. The increase in the number of polymorphisms analyzed by mass spectrometry in this gene allowed for a more accurate characterization of the genotype of these patients.

The two discordant cases for *CYP2B6* were patients identified as intermediate metabolizers and later categorized as poor, whereas the other patient was an efficient metabolizer. In both cases, it was due to incidents that affected the traceability of the results.

The only discordant case for *CYP2C9* was due to a variant associated with decreased enzyme activity in the patient (rs7900194, *8), which was only studied by the second genetic characterization method, mass spectrometry.

Of the four patients whose result was discordant for *CYP2C19*, in two of them, the variants found did not induce modifications of the phenotype. The other two, reported as efficient metabolizers, were finally classified as ultrarapid due to identifying the rs12248560, *17 allele, in heterozygosis.

Only two of the six patients with discordant results for *CYP2D6* changed their phenotype and this was due to the increase in the variants analyzed. One of the patients changed from an efficient metabolizer to an intermediate metabolizer, while the second patient changed from an intermediate to an efficient metabolizer. In addition, CNV and hybrids of *CYP2D6* could be simultaneously detected.

It is interesting to notice that in the case of *CYP3A4*, only the presence of the rs2740574 (*1B) mutation was analyzed initially, while in the second type of genotyping, the rs35599367 (*22) variant was studied. The nine discordant patients initially presented the rs2740574 (*1B) variant, but with mass spectrometry, they presented the second variant, rs35599367 (*22), although no modification of the predicted enzyme profile was detected.

Finally, as with the *CYP3A5* gene, the only patient whose result was discordant for *ABCB1* was considered an intermediate metabolizer, changing to a poor metabolizer by identifying the variant in homozygosis.

All of the discordant cases were clinically reported according to the new results to improve the clinical management of every patient. Therefore, 3% of all of the replicates performed were subject to change in the genotyping results due to technological improvements.

## 3. Discussion

### 3.1. Clinical Referral Service

Polypharmacy is a risk factor for the occurrence of adverse drug events, and pharmacogenetics is currently a great tool that allows healthcare professionals to improve the management of treatment prescriptions by identifying and predicting potential interactions. However, most clinical services do not routinely use it [5]. Traditionally, pharmacogenetics has been oriented to finding specific biomarkers for specific drugs individually considered; however, most patients are treated in polypharmacy regimes. In order to approach pharmacogenetics in real-world situations, a decade ago, we designed 5SPM to consider the complete spectrum of patient prescriptions simultaneously. Since then, we have used this methodology in our routine practice with clear advantages in clinical and economic aspects [9,12,39].

The service that most requested this methodology was Psychiatry, which sent three-quarters of the patients under study. The significant familiarity of these professionals with the potential of this pharmacogenetic approach and Psychiatry being one of the specialties most probed to polypharmacy favor this fact [40,41]. The management of these patients has changed in recent years. In our population, this is also due to the impact of the 5SPM model on the outcome of patients. The fifth step of the model includes the re-evaluation of the intervention. From the clinical point of view, we identified that the prescription of oral drugs whose metabolism was related to enzymes altered in the study population was reduced by between 20 and 100% after the application of the model, with a reduction in the mean dose of antipsychotics to approximately 50%, for example [39]. The application of the 5SPM model helped significantly reduce pharmacologic interactions in our patients by decreasing polytherapy, preventing side effects, and increasing adherence to treatment, with a consequent positive effect when assessing therapeutic success. Nowadays, pharmacogenetic analysis is recommended to be requested before treatment is initiated in order to avoid the occurrence of adverse effects or treatment ineffectiveness.

Certain services, such as Hematology [42] or Oncology [43,44,45], could greatly benefit from the application of these techniques since they use a large number of drugs concomitantly, including widely known treatments such as tamoxifen, which has been used for three decades in the treatment of breast cancer [46], one of the most prevalent pathologies in today’s society. These services now refer an increasing number of patients, indicating developments in the clinical management of patients.

However, although not all healthcare professionals in all specialties are familiar with these techniques, many have a positive attitude toward their application, which allows us to be broadly optimistic about their spread. Therefore, it is necessary to train both current and future generations [47].

### 3.2. Pharmacogenetic Analysis

In most studied genes, there were no significant differences in the allelic frequency of variants regardless of the methodology employed for genotyping. The results associated with the *CYP3A4* and *CYP3A5* genes are particularly noteworthy for variants associated with decreased enzyme activity. In the case of the *CYP3A4* gene, we observe that the allelic and genotypic frequencies of the variant that predict a decrease in enzyme activity were lower than the mean of the rest of the genes. In contrast, the alleles associated with *CYP3A5* decreased activity are markedly higher than in the other genes, affecting almost the entire population studied. A deficiency in this metabolic pathway renders it highly susceptible to drug interactions, particularly in individuals undergoing prolonged corticosteroid and immunosuppressant therapy [48].

In the case of *ABCB1*, we can also find relatively high allelic and genotypic frequencies of variants that produce a decrease in enzymatic activity, affecting more than half of the population studied. It is particularly relevant given that it is a transporter responsible for eliminating waste accumulated in the hepatocytes; so, a deficit in function can lead to drug accumulation that harms patient health [49,50,51].

*CYP2C9* represents approximately 50% of CYP2C subfamily expression [52], metabolizing around 15% of the drugs corresponding to the entire subfamily, which in turn metabolizes 20% of all drugs, especially non-steroidal anti-inflammatory drugs (NSAIDs) [53]. Likewise, it is also responsible for metabolizing certain organic compounds such as weakly acid molecules [54], so its genotyping would allow us to know their physiological behavior, which is especially useful given that almost 40% of the population studied has some polymorphism responsible for a decrease in enzyme activity.

In the case of *CYP2C19*, half of the population has either a variant of increased enzyme activity or a variant of decreased enzyme activity. This enzyme is used in Clopidogrel [55] or Selective Serotonin Reuptake Inhibitors (SSRIs). For this type of medication, the enzyme encoded by *CYP2B6* is also involved [56], and it is necessary to take into account that half of the population also has decreased enzyme activity, which makes it more complicated to adjust therapy metabolized by these pathways before genetic characterization [57,58,59].

Among the results obtained by both techniques for the gene *CYP1A2*, although more than half of the patients showed inducibility of enzymatic activity with both techniques, there are significant differences, given that more variants were analyzed by mass spectrometry, thus showing that there is a higher percentage of patients affected by susceptibility to inducibility of the metabolization pathway. Given that tobacco [22] and caffeine [23,24,25] are widely consumed in our society, it is interesting to know the metabolizing capacity of this enzyme to adjust treatments, both regularly and in hospitalized patients.

*CYP2D6*, one of the critical genes in pharmacogenetics, is responsible for the metabolism of approximately 20% of medication [60] mainly due to the existence of an innumerable interindividual variability, its high polymorphism, and the complexity of interpretation of the results [61,62], which has been improving in recent times thanks to the development of techniques such as the Cyrius tool [63], which improves the accuracy of sequencing and differentiation of *CYP2D6* and *CYP2D7*. Therapy with *CYP2D6* substrates can be complex, not only due to genetic variation but also due to drug–drug interactions. It is also essential to be aware of the phenocopy phenomenon [40], given that intermediate metabolizers sometimes behave as deficient when drugs are combined with *CYP2D6* inhibitors, implying that half of our patients have some form of decreased enzyme activity.

### 3.3. Comparison of Genetic Characterization Methods

According to the technology used for genotyping, 95.7% of the results were concordant, while 4.3% were discordant in our comparative study. Most of these discordant cases were due to improved technologies used in phenotyping, as the increased number of variants studied provided more information regarding the result obtained. It is therefore necessary to consider the possible need to review certain studies over time if the patient’s suitability for treatment has not been satisfactorily achieved, considering the economic upheaval that this may entail [64]. In our experience, the best performance was obtained through mass spectrometry, allowing for the simultaneous identification of more relevant genotypes, including the characterization of CNVs and complex hybrids. In addition, in our experience, this technology was not more expensive than the others, but it depends on the commercial presentations and combinations of the number of rs and samples included in each assay. However, it should be considered that specific training and competence are required for the technical management of mass arrays. This study did not include NGS due to inherent problems approaching complex structural variants that make validation difficult. However, introducing technology based on long reads could help solve these limitations.

We detected a traceability incident in four cases, <0.1% of the determinations, corresponding to 0.8% of patients, which underscores the need to improve the automatization in genetic laboratories to minimize incidents, especially regarding the transcription of information. Laboratory information management systems (LIMSs) are essential for providing data tracking support and avoiding potential errors from manual intervention. In addition, a regulatory framework, such as accreditation, is highly recommended to guarantee appropriate quality control of the procedures in clinical laboratories.

It is important to note that certain limitations were encountered in conducting this study. The sample of patients received was not homogeneous; proportionally, many patients were referred by the Psychiatry department. Likewise, not all patients referred to the Pharmacogenetics Unit could be studied by both techniques due to a lack of genetic material. In addition, specific techniques, such as microarrays, were commercially discontinued during this study. The limitations regarding NGS have been previously described. Concerning the interpretation of the results, it must be considered that the genotypes obtained can be deduced from the variants analyzed, and therefore, there is a possibility that other alleles may be present. In addition, the different techniques used, as we have seen, can detect different alleles. On the other hand, although training in interpreting such results is increasing, it is necessary to train the laboratory staff well so they know how to transmit the information clearly and concisely to the petitioners. Both the interpretation of the phenotypic prediction and its implication for the pharmacological situation are critical aspects of the application of pharmacogenetics.

In conclusion, including mass spectrometry in the laboratory has allowed for a more comprehensive approach to the pharmacogenetic situation of our polymedicated patients. This technology has facilitated the simultaneous analysis of larger and more complete panels of metabolizing enzymes, giving us a more comprehensive view of these studies’ complexity. Genetic characterization techniques have been recently improved by leaps and bounds [65]. This fact, added to the accessibility of genetic panels, is increasingly cheaper and allows for their clinical implementation more efficiently. Genetic services are going to be used more and more frequently, with Spain being one of the pioneering health systems in the use of pharmacogenetics in routine healthcare [66].

## 4. Material and Method

### 4.1. Subjects and Study Design

This study focuses on 596 patients referred to the Pharmacogenetics Unit of the University Hospital of Salamanca between 2013 and 2021. All procedures were carried out according to the relevant guidelines and regulations. Written informed consent was obtained from all patients according to the recommendations of the Ethics Committee of the University Hospital of Salamanca. This study was approved by this committee with Ref Cod. CEIm PI 2021 08 866.

Of these 596 patients, 523 had the concomitant presence of at least two drugs and other inclusion criteria such as adverse effects, intolerance to treatment, partial response to treatment, or therapeutic failure. Of the remaining patients, 29 were referred before the initiation of their treatment, while 44 were referred without knowing their current treatment.

Our Pharmacogenetic model called the Five-Step Precision Medicine (5SPM) model [9,12,39] was applied to all patients, allowing them to improve their response to treatment. This model consists of the following:

I. Step 1: clinical, epidemiological, and therapeutic data collection;
II. Step 2: the prediction of drug interactions and pharmacokinetic-specific pathways;
III. Step 3: pharmacogenetic analysis of selected genes;
IV. Step 4: rationalized PGx-guided adjustments of drug therapy;
V. Step 5: an assessment of the intervention and model re-evaluation.

### 4.2. Genotyping Techniques

This study was developed in an accredited laboratory according to ISO15189 [67], including participation in specific National Proficiency Testing for Pharmacogenetics. Aspects related to the quality of data and procedures have been carefully addressed, including precision, accuracy, consistency, and validity.

Different techniques were used for the genotyping of cytochrome P450 superfamily genes *CYP1A2*, *CYP2B6*, *CYP2C9*, *CYP2C19*, *CYP2D6*, *CYP3A4*, and *CP3A5* and the transmembrane transporter ATP binding cassette transporter A1 (*ABCA1*), after the extraction of genomic DNA from peripheral blood using an automated MagNa Pure Compact system (Roche Diagnostics, Roche Applied Science, IN, USA) or Qiacube system (Qiagen, Hilden, Germany).

#### 4.2.1. Real-Time PCR High-Resolution Melting

This technique consists of determining mutations by using specific real-time duplex PCR and detection with a FRET (fluorescence resonance energy transfer) hybridization probe, with specific primers for the allelic discrimination of genetic variants, based on energy emission transfer technology and analysis by melting curves.

This allelic discrimination was performed by high-resolution melting (HRM) through the analysis of dissociation curves, also called melting curves, using LightCycler 2 and LightcCyler 480 System II platforms (Roche Molecular Diagnostics, Pleasanton, CA, USA).

Detecting the differences in the curves due to a change in a single base in the amplified DNA sequence allows for the differentiation of a specific polymorphism. It also allows for the distinction between homozygosis, in which only one melting curve will be observed, and heterozygosis, in which two different curves will be reflected.

The LightCycler FastStart DNA MasterHybProbe kit’s reagents were used according to the manufacturing recommendations. Both positive and negative controls were used in all cases and handled in a biological safety flow cabinet to avoid potential contaminations during handling.

#### 4.2.2. Microarray Technique

For the analysis of the *CYP2D6* gene, semiquantitative PCR amplification was performed, followed by variant detection based on the direct array hybridization technique with Infiniti Plus technology (Autogenomics, Carlsbad, CA, USA). This method uses target signal amplification to measure fluorescent signals from labeled DNA targets hybridized to the microarray.

The Infiniti Plus *CYP2D6* assays were based on the amplification of purified DNA by PCR, the incorporation of the fluorescent marker using the analyte-specific primer extension (ASPE), and the hybridization of ASPE primers to a microarray followed by washing. Subsequently, the microarray had to be scanned to detect fluorescence signals and analyze the data obtained.

#### 4.2.3. MALDI-TOF Mass Spectrometry

The analysis of SNPs by mass spectrometry MALDI-TOF (Matrix-Assisted Laser Desorption/Ionization and Time-Of-Flight detector) was performed using the MassARRAY System (Agena Bioscience, San Diego, CA, USA). The workflow couples mass spectrometry with end-point PCR followed by a single-base extension reaction, enabling highly multiplexed reactions under universal cycling conditions to provide a quick genetic analysis.

Following the manufacturer’s recommendations, we used the AgenaBioscience pre-designed panel called VeriDose Core Panel for SNPs and VeriDose *CYP2D6* CNVPanel Set for *CYP2D6* CNVs.

Finally, a comparison of the results obtained with the different employed techniques was carried out. Table 4 and Table 5 detail the variants analyzed for each gene according to the technology used.

It should be noted that the difference between the variants analyzed by each technique was due to the commercial kit used for each analysis.

The information from PharmGKB [101,102] and PharmVar [68] was used to study the functional categorization of variants according to the mutations analyzed, especially information concerning *CYP2D6* diplotype–phenotype conversion [103].

### 4.3. Statistical Analysis

The data were collected using Microsoft Excel and Microsoft Access (www.microsoft.com), allowing for the classification of patients according to age, sex, the reason for requesting the pharmacogenetic study, the clinical service of origin, and the results of the pharmacogenetic study. A pseudonymization procedure was also carried out for all of the databases.

All statistical analyses were performed using Statistical Package for the Social Sciences (SPSS) version 21 (IBM Chicago, IL, USA). Descriptive statistics was used to determine central tendency and dispersion. The normality of the distribution was assessed using the Kolmogorov–Smirnov test or Shapiro–Wilk test. The Fisher test, Montecarlo test, T-Student test, ANOVA, or χ^2^ of Pearson was carried out for the bivariate analysis. The equality of variances was ensured by using the Levene test. Quality control correction for multiple comparations, false-positive report probability (FPRP), and statistical power were also calculated.

## 5. Conclusions

The results of our comparison studies revealed the advantages of mass spectrometry over the other methodologies analyzed, the importance of updating the laboratory’s pharmacogenetic methodology to match it with the identification of new variants with clinical relevance, and the need to introduce information systems with direct dumping of data that minimize the risks of decreasing the traceability of the results.

## Figures and Tables

**Table 1:** The distribution of the allelic frequencies of the different genes studied according to the genetic characterization method.

Gene	Allele	Predicted Enzyme Activity	Real-Time PCR			MALDI-TOF		
			N	Studied Population Frequency (%)	Number of Alleles	N	Studied Population Frequency (%)	Number of Alleles
*CYP1A2*	*1A	Normal	460	65.65	302	394	53.81	212
	*1F	Inducible		34.35	158		45.18	178
	*1K	Decreased		-	-		0.25	1
	*1L	Decreased		-	-		0.76	3
*CYP2B6*	*1	Normal	566	72.79	412	408	67.89	277
	*6	Decreased		27.21	154		32.11	131
*CYP2C9*	*1	Normal	636	78.30	498	404	75.49	305
	*2	Decreased		14.78	94		17.33	70
	*3	Decreased		6.92	44		7.18	29
*CYP2C19*	*1	Normal	690	485	70.29	404	70.55	285
	*2	Decreased		103	14.93		11.88	48
	*3	Decreased		-	-		0.25	1
	*4	Decreased		-	-		0.49	2
	*17	Increased		102	14.78		16.83	68
*CYP3A4*	*1A	Normal	688	655	95.20	402	92.54	372
	*1B	Decreased		33	4.80		5.47	22
	*2	Decreased		-	-		0.50	2
	*22	Decreased		-	-		1.49	6
*CYP3A5*	*1	Normal	662	91.84	608	404	5.45	22
	*3	Decreased		8.16	54		93.07	376
	*6	Decreased		-	-		1.48	6
*ABCB1*	C	Normal	634	55.68	353	402	55.49	211
	T	Decreased		44.32	281		47.51	191
**Gene**	**Allele**	**Predicted Enzyme Activity**	**Microarray Technique**			**MALDI-TOF**		
			**N**	**Studied Population Frequency (%)**	**Number of Alleles**	**N**	**Studied Population Frequency (%)**	**Number of Alleles**
*CYP2D6*	*1	Normal	672	38.84	261	378	34.13	129
	*2	Normal		27.98	188		22.75	86
	*3	None		1.64	11		1.32	5
	*4	None		19.20	129		21.69	82
	*5	None		1.93	13		1.59	6
	*6	None		1.49	10		0.53	2
	*7	None		0.15	1		-	-
	*9	Decreased		3.13	21		4.50	17
	*10	Decreased		1.19	8		0.53	2
	*12	None		0.15	1		-	-
	*17	Decreased		0.45	3		-	-
	*35	Normal		0.45	3		-	-
	*41	Decreased		2.53	17		7.67	29
	*83	Uncertain		-	-		0.26	1
	*1XN	Increased		0.15	1		1.59	6
	*2XN	Increased		0.45	3		0.79	3
	*4XN	None		0.30	2		2.38	9
	*41XN	Decreased		-	-		0.26	1

**Table 2:** The distribution of genotype frequencies of the different genes studied according to the genetic characterization method.

Gene	Genotype	Predicted Phenotype	Real-Time PCR			MALDI-TOF		
			Frequency (%)	N	Total N	Frequency (%)	N	Total N
*CYP1A2*	*1A/*1A	EM	44.35	102	230	32.99	65	197
	*1A/*1F	INDUCIBLE	42.61	98		41.62	82	
	*1F/*1F	INDUCIBLE	13.04	30		23.35	46	
	*1F/*1K	IM	-	-		0.51	1	
	*1F/*1L	INDUCIBLE	-	-		1.52	3	
*CYP2B6*	*1/*1	EM	53.36	151	283	46.08	94	204
	*1/*6	IM	38.87	110		43.63	89	
	*6/*6	PM	7.77	22		10.29	21	
*CYP2C9*	*1/*1	EM	61.63	196	310	56.93	115	202
	*1/*2	IM	22.96	73		25.74	52	
	*1/*3	IM	10.38	33		11.39	23	
	*2/*2	PM	1.89	6		3.47	7	
	*2/*3	PM	2.83	9		1.98	4	
	*3/*3	PM	0.31	1		0.49	1	
*CYP2C19*	*1/*1	EM	47.24	163	345	47.03	95	202
	*1/*2	IM	20.29	70		17.82	36	
	*1/*3	IM	-	-		0.49	1	
	*1/*4	IM	-	-		0.99	2	
	*1/*17	UM	25.80	89		27.72	56	
	*2/*2	PM	2.90	10		0.49	1	
	*2/*17	IM	3.77	13		4.95	10	
	*17/*17	UM	-	-		0.49	1	
*CYP3A4*	*1A/*1A	EM	90.99	313	344	86.07	173	201
	*1A/*1B	IM	8.43	29		8.96	18	
	*1B/*1B	PM	0.58	2		0.99	2	
	*1A/*2	IM	-	-		0.99	2	
	*1A/*22	IM	-	-		2.99	6	
*CYP3A5*	*1/*1	EM	0.91	3	331	-	-	202
	*1/*3	IM	14.50	48		10.39	21	
	*1/*6	IM	84.59	289		0.50	1	
	*3/*3	PM	-	-		86.63	175	
	*3/*6	PM	-	-		2.48	5	
*ABCB1*	C/C	EM	31.55	100	317	54	26.87	201
	C/T	IM	48.26	153		103	51.24	
	T/T	PM	20.19	64		44	21.89	
**Gene**	**Genotype**	**Predicted Phenotype**	**Microarray Technique**			**MALDI-TOF**		
			**Frequency (%)**	**N (336)**		**Frequency (%)**	**N (189)**	
*CYP2D6*	*1/*1	EM	16.07	54		13.22	25	
	*1/*2	EM	21.13	71		15.24	29	
	*1/*3	IM	1.79	6		1.59	3	
	*1/*4	IM	14.88	50		13.76	26	
	*1/*5	IM	1.19	4		0.53	1	
	*1/*6	IM	0.89	3		1.05	2	
	*1/*7	IM	0.20	1		-	-	
	*1/*9	EM	2.08	7		2.12	4	
	*1/*10	EM	0.89	3		0.53	1	
	*1/*35	EM	0.30	1		-	-	
	*1/*41	EM	1.49	5		4.23	8	
	*1/*1XN	UM	-	-		2.12	4	
	*1/*2XN	UM	0.60	2		0.53	1	
	*1/*41XN	EM	-	-		0.53	1	
	*2/*2	EM	7.74	26		3.70	7	
	*2/*3	IM	1.19	4		0.53	1	
	*2/*4	IM	12.20	41		14.81	28	
	*2/*5	IM	0.89	3		1.06	2	
	*2/*6	IM	0.89	3		-	-	
	*2/*9	EM	2.38	8		2.12	4	
	*2/*10	EM	0.30	1		-	-	
	*2/*17	EM	0.30	1		-	-	
	*2/*41	EM	0.89	3		3.18	6	
	*2/*83	IM	-	-		0.53	1	
	*2/*4XN	IM	0.30	1		0.53	1	
	*3/*4	PM	-	-		0.53	1	
	*3/*9	IM	0.30	1		-	-	
	*4/*4	PM	3.87	13		3.18	6	
	*4/*5	PM	0.30	1		0.53	1	
	*4/*9	IM	0.60	2		1.59	3	
	*4/*10	IM	0.89	3		-	-	
	*4/*35	IM	0.30	1		-	-	
	*4/*41	IM	1.19	4		2.65	5	
	*4/*4XN	PM	0.30	1		2.12	4	
	*5/*5	PM	0.60	2		-	-	
	*5/*6	PM	0.30	1		-	-	
	*5/*41	IM	-	-		1.06	2	
	*6/*9	IM	0.30	1		-	-	
	*6/*41	IM	0.30	1		-	-	
	*9/*9	IM	-	-		1.06	2	
	*9/*41	IM	0.30	1		-	-	
	*10/*12	IM	0.30	1		-	-	
	*17/*17	IM	0.30	1		-	-	
	*35/*41	EM	0.30	1		-	-	
	*41/*41	IM	0.30	1		1.06	2	
	*1XN/*6	EM/UM	0.30	1		-	-	
	*1XN/*9	EM/UM	-	-		0.53	1	
	*1XN/*41	EM/UM	-	-		0.53	1	
	*2XN/*4	EM/UM	-	-		1.06	2	
	*2XN/*9	EM/UM	0.30	1				
	*4XN/*9	IM	-	-		0.53	1	
	*4XN/*41	IM	-	-		1.59	3	

PM: poor metabolizer, EM: efficient metabolizer, IM: intermediate metabolizer.

**Table 3:** The results obtained in the comparison of characterization methods according to the genes studied.

Gene	N	Concordance	Change Phenotype ^1^
*CYP1A2*	48	46 (95.8%)	2/2
*CYP2B6*	64	62 (96.9%)	2/2
*CYP2C9*	78	77 (98.7%)	1/1
*CYP2C19*	98	94 (95.9%)	2/4
*CYP2D6*	97	91 (93.8%)	2/8
*CYP3A4*	97	88 (90.7%)	0/9
*CYP3A5*	93	92 (98.9%)	1/1
*ABCB1*	82	81 (98.8%)	1/1

^1^ Variations in genotype implying variations in phenotype.

**Table 4:** Variants analyzed for each gene comparing real-time PCR high-resolution melting and MALDI-TOF mass spectrometry techniques.

Gene	Genotyping Technique		Function	Reference [68]
	Real-Time PCR	MALDI-TOF		
*CYP1A2*		rs2069514	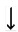	[69]
	rs762551	rs762551	Highly inducible	[70]
		rs12720461	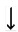	[71]
		rs56107638	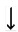	[72,73]
		rs72547513	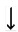	[74]
*CYP2B6*		rs28399499	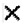	[75]
	rs3745274	rs3745274	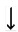	[76]
*CYP2C9*	rs1799853	rs1799853	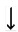	[77,78,79,80]
	rs1057910	rs1057910	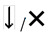	[81,82]
		rs56165452	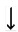	[83]
		rs28371686	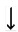	[57]
		rs9332131	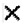	[57]
		rs7900194	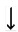	[57]
		rs28371685	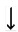	[57]
		rs9332239	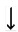	[57]
		rs72558187	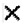	[57]
		rs72558188	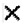	[57]
		rs72558190	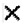	[57]
*CYP2C19*	rs4244285	rs4244285	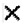	[58]
	rs4986893	rs4986893	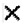	[58]
	rs12248560	rs12248560	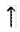	[84]
		rs28399504	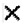	[85]
		rs56337013	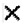	[86]
		rs72552267	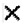	[86]
		rs72558186	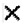	[86]
		rs41291556	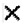	[86]
*CYP3A4*	rs2740574		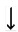	[48,87,88]
		rs55785340	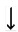	[89]
		rs4987161	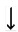	[90,91]
		rs35599367	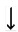	[92]
*CYP3A5*		rs28365083	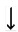	[93,94,95]
	rs776746	rs776746	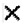	[96]
		rs10264272	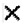	[97]
		rs41303343	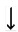	[98]
*ABCB1*	rs1045642	rs1045642	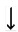	[99,100]

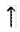
 increased enzyme function; 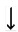 decreased enzyme function; 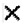 no enzyme function.

**Table 5:** Variants analyzed for *CYP2D6* gene comparing microarray and MALDI-TOF mass spectrometry techniques.

GENE *CYP2D6*	
Microarray Technique	MALDI-TOF
	rs1135840
rs1080985	
rs1065852	rs1065852
rs28371706	
rs5030655	rs5030655
rs5030865	rs5030865
rs5030865	rs5030865
rs3892097	rs3892097
rs5030862	rs5030862
rs61736512	
rs28371725	rs28371725
rs35742686	rs35742686
rs5030656	rs5030656
rs16947	rs16947
rs5030867	rs5030867
*5del	
dup4125_4133	dup4125_4133
	rs5030863
	rs72549357
	rs28371706
	rs72549353
	rs72549354
	rs59421388
	rs28371735

## Data Availability

The original contributions presented in the study are included in the article, further inquiries can be directed to the corresponding author.
